# Exploration of psychosocial and environmental factors that could improve the consumption of iron-rich foods among urban Senegalese adolescent girls

**DOI:** 10.11604/pamj.2025.50.29.36376

**Published:** 2025-01-16

**Authors:** Aminata Ndéné Ndiaye, Isabelle Galibois, Sonia Blaney

**Affiliations:** 1 Laval University, Quebec City, Quebec, G1V 0A6, Canada,; 2 Moncton University, Moncton, New Brunswick, E1A 3E9, Canada

**Keywords:** Iron-rich foods, theory of planned behavior, adolescent girls, individual and environmental factors, Senegal

## Abstract

**Introduction:**

anaemia remains a public health issue among adolescent Senegalese girls, and one cause is the low consumption of iron-rich foods. This study used the extended model of the Theory of Planned Behavior (TPB) to explore psychosocial factors and environmental barriers that may influence the daily consumption of iron-rich foods (IRF) among urban Senegalese adolescent girls.

**Methods:**

a cross-sectional survey was conducted among 136 girls (13-18 years). Salient beliefs related to each construct of the theory were identified. Using this information, a questionnaire was developed to collect data on each construct and the intention to consume IRF daily.

**Results:**

on a scale of -2 to 2, the mean score of the intention was 1.39 ± 0.74 while average scores of direct constructs were 1.60 ± 0.89 for the attitude, 1.29 ± 0.84, for the subjective norm, 0.82 ± 0.91 for the perceived behavioral control, and -0.14 ± 0.86 for the environmental barriers. Overall, 34% of girls reported that it was likely that implementing the behavior would make them gain weight while more than 80% stated that their father/mother/sisters would approve the behavior. Also, 38% of girls did not feel able to perform the behavior if they were not capable of preparing IRF themselves. Half agreed that the high price of these foods was a barrier to their consumption.

**Conclusion:**

most adolescent girls intend to consume IRF. To operationalize the intention into a concrete behavior, interventions increasing self-efficacy and improving knowledge about IRF, and their affordability and accessibility could be relevant.

## Introduction

Anaemia is a global public health problem that affects about one-third of the world's population, with well-documented human health and economic consequences [1]. Adolescent girls in low- and middle-income countries/LMICs appear to be particularly at risk of iron deficiency anaemia due to their increased iron requirements combined with limited dietary intakes, high rates of infections and parasitic infestations, as well as the occurrence of early pregnancy [2]. Socioeconomic, behavioral, and environmental factors may also increase their vulnerability to anaemia given their influence on the consumption of iron-rich foods/IRF [1]. In Senegal, the situation of anaemia among adolescent girls has remained unchanged over the last decade, with 56% of them reported as being anaemic in 2010-2011 [3] and 57% in 2017 [4]. Similarly, to most LMICs, anaemia in Senegal is mainly from the nutritional origin, resulting from a low intake of bioavailable iron [5]. Indeed, the diet of most populations in LMICs, especially those in the sub-Saharan African region, is characterized by a limited food diversity and high consumption of plant-based foods, low in bioavailable iron [6,7]. This poor diet quality is also observed in adolescents [6].

Current strategies implemented in Senegal to reduce and control iron deficiency and iron deficiency anaemia are large-scale iron/folic acid fortification of wheat flour and iron and folic acid/IFA supplementation for pregnant women. Regarding flour fortification, its contribution to micronutrient intake, especially iron, seems insufficient [8]. This is mainly due to the use of low bioavailability forms of iron, insufficient consumption, and inadequate fortification levels [9]. Concerning IFA supplementation, this strategy was initiated in 2017 in adolescent girls living in seven regions of Senegal, among which the region of Dakar. However, it should be noted that iron supplementation programs in LMICs generally face logistical and economic constraints that limit the supply and distribution of supplements and coverage of at-risk populations [10,11]. Otherwise, strategies focusing on the increase of iron intake through dietary diversification and modification among adolescent girls are scarce, although they have been associated with continued improvements in iron status among women of reproductive age and children in LMICs [12-14]. We previously documented the consumption of IRF in urban Senegalese adolescent girls [15]. Results have shown that 83% of them had a daily intake below the recommendation of 84g of animal protein foods from the EAT-Lancet Commission on healthy diets from sustainable food systems [16], which is worrisome. The diet of Senegalese girls appears to be conducive to iron deficiency, hence justifying the importance of studying factors underlying low IRF consumption to define effective interventions through diet diversification and modification to improve the situation [17].

Psychosocial theories could be of substantial assistance in understanding mechanisms underlying behavior change and identifying potential influences on eating behaviors [18-20]. Moreover, their relevance for defining effective interventions for behavior change has been demonstrated in various studies [21-23]. Some of these theories were recognized as well adapted to investigate factors associated with adolescent behaviors [24]. One of them, the Theory of Planned Behavior/TPB, has been used in several settings to study what influences adolescents` eating habits [25-28]. To get a better understanding of the range of variables to consider when acting on health behavior, Godin *et al*. [29] have proposed an extended model of TPB that incorporates individual and environmental elements as determinants of the intention to engage in a behavior and its actual adoption. Based on the extended TPB [30,31], this study aimed to explore individual and environmental factors that can potentially influence the daily consumption of IRF among urban Senegalese adolescent girls aged 13-18 years old. The 13-18 age group, which corresponds to college students´ ages, was targeted to facilitate study logistics. More specifically, this study sought to answer the following research questions: a) To what extent do urban Senegalese adolescent girls intend to consume IRF? b) What psychosocial factors (i.e. attitude, subjective norm, perception of behavioral control, and their respective set of beliefs) are likely to influence their daily consumption of IRF? c) What environmental factors are likely to influence their daily consumption of IRF?

## Methods

**Study setting:** the study was carried out in the Dakar region of Senegal, a country located in the extreme west of the African continent. Senegal covers an area of 196,722 km^2^ and has a wide opening onto the Atlantic Ocean with 700 kilometers of coastline [30]. In 2023, Senegal's population was estimated at 18,126,390, characterized by extreme youth (average age 19). The population density is 95 inhabitants/km^2^, with strong disparities between regional administrative entities. The Dakar region, home to the capital, has 4 004 426 inhabitants, or 22% of the population, and is administratively subdivided into four departments: Dakar, Pikine, Guédiawaye, and Rufisque [31]. Senegal has a high prevalence of anaemia among women of reproductive age, 52.7% in 2019 [32]. Dakar is one of the Senegalese regions with the highest prevalence of anemia: in 2011, 58.5% of women of reproductive age suffered from anaemia, compared with 54.3% at the national level [3].

**Study design and target population:** this was a cross-sectional survey targeting adolescent girls from two colleges in the city of Dakar, Senegal. It was part of a three-part research that also aimed at assessing the intake of IRF [15] in girls aged 13-18 years old and at defining, implementing, and assessing the impact of interventions to increase IRF intake [33]. The two colleges were selected on a purposive basis and, in each of them, all classes attended by girls aged 13-18 years were selected. In every class, all girls were invited to participate. Recruitment took place at the colleges, using the enrolment registers. In each college, the identified participants were called into a classroom where the study was presented, and where they received information about its purpose, the data to be collected, and the issues of anonymity and voluntaries. Those willing to participate were given a consent form for their parents/guardians to sign.

**Study sample size:** the required number of observation units was calculated using the G*power 3.1.9.2 software, considering the following parameters: statistical power of 80%; 95% accuracy; an effect size of 0.5 and a non-response rate of 5%. Thus, a sample comprising two groups of 67 adolescents each was necessary to carry out the project.

### Variables

**Behavior of interest:** in this research, the behavior of interest was the consumption of IRF which included red meat (beef, mutton, lamb), poultry, fish, shellfish, offal, and eggs, according to the list currently used in national demographic and health surveys [4].

**Theoretical framework:** according to the TPB, one will adopt a behavior if she/he has the intention to perform it. In turn, the intention is determined by three constructs namely the attitude (ATT), the subjective norm (SN), and the perceived behavioral control (PBC) [34]. ATT towards a behavior refers to an individual's perception of the consequences of adopting it. The subjective norm (SN) represents the perceived social pressure to adopt a given behavior while PBC refers to the individual's evaluation of his/her ability to implement the behavior [34]. Moreover, ATT is determined by two indirect constructs: one's beliefs about outcomes of performing a behavior (or: behavioral beliefs), weighted by the evaluation of those outcomes. The subjective norm (SN) is determined by individual beliefs about whether important individuals or groups will approve or disapprove of performing a behavior (or: normative beliefs), weighted by his/her motivation to comply. PBC is also determined by two indirect constructs: control beliefs, weighted by the perceived power of each control factor to facilitate or not the behavior [35]. The extended TPB suggests that these three constructs can be influenced by external factors that include individual characteristics (e.g, gender, age, socioeconomic status, education level, etc.) and environmental characteristics (social or physical). These various external variables can also impact intention and/or behavior [29].

**Data sources:** the data collection was conducted from March to April 2019. A key step of this research was to develop a questionnaire to gather information on each component of the theoretical framework namely psychosocial factors (ATT, SN, PBC, and their respective related set of beliefs), some environmental factors, and the intention to consume 85g of IRF daily which was the behavior of interest in the current study. Plastic foods and real IRF representing an 85-g portion were used so that girls could visualize this serving size. The first step in the development of the questionnaire was the identification of salient beliefs associated with each of the individual constructs of the TPB model, as well as the investigation of some environmental factors that may promote or hinder the consumption of IRF. To do so, three focus group discussions (FGD) of about 60-90 minutes were held in French with ten (10) girls each. A semi-structured interview guide was used to collect information on the above components. Data on the following were gathered: a) advantages and disadvantages associated with the daily consumption of at least 85g of IRF (red meat, poultry, fish, shellfish, offal, and eggs), b) persons or groups of persons important who would approve or disapprove of their daily consumption of at least 85g of IRF, c) individual barriers and facilitators related to the daily consumption of IRF, and d) elements related to the adolescent girls' environment that could influence their daily consumption of at least 85g of IRF. For each of these questions, every individual's personal belief was listed. Thereafter, results were compiled. Beliefs expressing the same idea were grouped, and the frequency of mention of each of these beliefs under each theme was computed. Beliefs that were mentioned by at least 20% of the girls (three FGD combined, N = 30) were retained and considered as the modal salient beliefs [36].

Subsequently, salient beliefs were used to formulate items of a questionnaire in French to be administered to girls to assess each component of the theoretical framework. Five-point Likert scales (with choices of responses ranging from 'strongly disapproved' to 'strongly approved' or from 'very unlikely' to 'very likely') were used to gather their responses to each item. In addition, the questionnaire included two items to assess the intention to adopt the targeted behavior. One item was worded to capture the intent while the other was worded to focus on self-prediction, as both of these concepts have been used to measure the intention in TPB studies [29,37]. Before its administration to the study sample, the questionnaire was pre-tested among two groups of five (5) adolescent girls from one college to verify their understanding of the questions and the proposed response choices on Likert scales. The final questionnaire composed of thirty-nine (39) items, which was a self-administered tool, was then filled by each adolescent in her classroom during a school day. Data on socio-economic characteristics of adolescent households were separately collected during interviews lasting from 45 to 60 minutes held in the Wolof language at home with the head of each participating adolescent's household, using the standardized and validated Senegal Demographic and Health Surveys questionnaire [3]. Information on household composition such as the age and gender of each member, as well as their level of education, was collected. In addition, data on household food security were collected using the Household Food Insecurity Access Scale (HFIAS) which has been used in Senegal [38].

**Statistical analysis:** for the self-administered questionnaire, scores ranging from -2 to 2 were assigned to response options on Likert scales based on the level of disagreement/unlikelihood to agreement/likelihood towards each statement. Except for ATT which was measured by a single item of the self-administered questionnaire, for each other direct construct (SN, PBC, environment) and indirect construct (behavioral beliefs, normative beliefs, and control beliefs) of the extended TPB model, a mean score was calculated for each adolescent by averaging her related item scores. Moreover, each household was categorized as food-secure, or mildly, moderately or severely food-insecure according to the classification method of Coates *et al*. [38].

**Sociodemographic data were grouped into categories:** descriptive analyses were performed: frequency distributions, means, and standard deviations were calculated. Data were analyzed with the SPSS software (Statistical Package for Social Sciences (SPSS), Version 21.0, Armonk, NY: IBM Corporation).

**Ethics:** the study was approved by the ethical committee of the National Health Research of Senegal 000106/MSAS/DPRS/CNERS and by the Research Ethics Committee of Laval University 2018-214/17-12-2018. Authorizations were also obtained from the Ministry of the National Education/School Medical Control Division, Dakar/Senegal. Written informed consent was obtained from the parent/tutor of each adolescent at the beginning of the study, and each girl also provided her written assent to participate.

## Results

Slightly more than half of adolescent girls were aged between 13 and 15 years and about 60% lived in male-headed households (Table 1). One out of two heads of household was above 50 years old, and 42% had no formal education. Half of households had more than eight members. Almost two thirds of households were food insecure. The mean score of the two items measuring adolescent girls' intention to consume at least 85g of IRF daily was 1.39 ± 0.74 out of a maximum of 2 (Table 2).

**Table 1:** characteristics of adolescent girls and their households (N = 136)

Characteristics	%	Mean ± SD
**Age of adolescent girls (years)**		15.0 ± 1.2
13 - 15	59	
16 - 18	41	
**Gender of head of household**		
Male	59	
Female	41	
**Education level of head of household:**		
None	42	
Primary incomplete/complete	23	
Secondary incomplete/complete	25	
Superior/university	8	
Other/not applicable	2	
**Age of head of household (years)**		53.1 ± 13.6
30 - 39	10.2	
40 - 49	27.7	
50 - 59	34.3	
60 - 69	16.8	
≥ 70	10.9	
**Number of persons in the household**		8.2 ± 3.7
2 - 7	49	
8 - 13	38	
14 - 30	13	
**Household food security**		
Food secure	37	
Mildly food insecure	17	
Moderately food insecure	17	
Severely food insecure	29	

**Table 2:** distributions (%) of girls on a five-response scale and mean scores (± standard deviation/SD) of their intention to consume at least 85g of iron-rich foods per day (N = 136)

Construct and items	% By five-response scale and score					Means (± SD)
Intention	Very improbable (-2)	Slightly improbable (-1)	Neither probable nor improbable (0)	Slightly probable (+1)	Very probable (+2)	
I intend to consume at least 85g of iron-rich foods per day	1.5	2.9	5.1	14.7	75.7	1.60 ± 0.84
I will eat at least 85g of iron-rich foods per day	2.2	7.4	5.9	38.2	46.3	1.19 ± 0.99
All						1.39 ± 0.74

**Attitude (direct construct) and behavioral beliefs:** the mean score of the direct measurement of ATT was 1.60 ± 0.89 while the average score on items related to behavioral beliefs was 1.25 ± 0.64 (Table 3). More than 85% of girls responded that it was likely or very likely that consuming at least 85g of IRF daily will allow them to be in good health, to grow well, to be more concentrated at school, and to prevent anaemia, while about 75% answered that it was likely or very likely that if they implement the behavior, they will be less tired and that it will prevent pregnancy complications. Otherwise, 35% answered that it was likely or very likely that eating at least 85g of IRF daily will make them gaining weight. Mean scores for all aforementioned items were above 1.00 except that on the item related to weight gain which was 0.00 (Table 3).

**Table 3:** distributions (%) of adolescent girls by item on a five-response scale and mean scores (± standard deviation/ SD) on direct and indirect constructs underlying the intention to consume at least 85 g of iron-rich foods per day (N=136)

Construct and items	% by five-responses scale and scores					Means (± SD)
ATT (direct construct)	Very unlikely (-2)	Slightly unlikely (-1)	Neither unlikely (0)	Slightly likely (+1)	Very likely (+2)	
For me, consuming at least 85g of IRF would be helpful	2.9	1.5	5.1	13.2	77.2	1.60 ± 0.89
Behavioral beliefs (indirect construct)						
If I consume at least 85g of IRFs, this will allow me...						
To be in good health	2.9	2.2	5.9	13.2	75.7	1.57 ± 0.92
To grow well	1.5	2.9	2.9	11.0	81.6	1.68 ± 0.79
To be less tired	4.4	3.7	15.4	27.9	48.5	1.12 ± 1.08
To be more concentrated at school	2.9	2.2	8.1	14.0	72.8	1.51 ± 0.95
To prevent pregnancy complications in the future	5.1	2.9	15.4	15.4	61.0	1.24 ± 1.14
To prevent anaemia	3.7	1.5	8.1	8.1	78.7	1.57 ± 0.97
To make up for the blood loss during menstruation	6.6	2.2	11.8	10.3	69.1	1.33 ± 1.18
To gain weight	19.1	14.7	31.6	16.9	17.6	0.00 ± 1.34
All						1.25 ± 0.64
**SN (direct construct)**	**Strongly disapprove**	**Slightly disapprove**	**Neither approve nor disapprove**	**Slightly approve**	**Strongly approve**	
Most people who are important to me would recommend that I consume at least 85g of iron-rich foods per day	6.6	0.7	5.1	23.5	64.0	1.38 ± 1.09
If I consumed at least 85g of iron-rich foods per day, most of the people who are important to me would strongly endorse it	5.1	6.6	7.4	27.9	52.9	1.17 ± 1.15
The people most important to me think I should be consuming at least 85g of iron-rich foods per day	4.4	2.9	13.2	13.2	66.2	1.34 ± 1.10
All						1.29 ± 0.84
Normative beliefs (indirect construct)						
Do you think the following people would approve that you consume at least 85g of iron-rich foods daily?						
Dad	1.5	2.9	7.4	14.7	73.5	1.56 ± 0.87
Mom	0.7	2.9	4.4	16.2	75.7	1.63 ± 0.77
Sisters	0.7	4.4	14.7	23.5	56.6	1.31 ± 0.93
Brothers	1.5	2.9	16.2	27.9	51.9	1.25 ± 0.93
Cousins (male)	4.2	4.4	27.2	16.9	47.1	0.98 ± 1.15
Cousins (female)	2.9	6.6	19.1	22.1	49.3	1.08 ± 1.10
Aunts	2.9	3.7	19.9	21.3	52.2	1.16 ± 1.06
Grandmothers	4.4	3.7	14.7	16.2	61.0	1.26 ± 1.12
Grandfathers	2.2	2.9	31.6	19.9	43.4	0.99 ± 1.04
Sandwich sellers	5.9	2.2	30.9	11.0	50.0	0.97 ± 1.04
All						1.22 ± 0.69

**Subjective norm (direct construct) and normative beliefs:** the mean score of the direct measurement of the SN was 1.29 ± 0.84 while that of the indirect measurement (normative beliefs) was 1.22 ± 0.09. Overall, over 80% of girls responded that their father, mother, and sisters would slightly or strongly approve if they consumed at least 85g of IRF daily. Other groups of people were mentioned by lesser proportions (Table 3).

**Perceived behavioral control (direct construct) and control beliefs:** overall, the mean score of the direct measurement of the PBC construct was 0.82 ± 0.91, while that of control beliefs (indirect measurement) was 0.01 ± 1.13 (Table 3.1). About 60% of girls responded that it was likely or very likely that eating at least 85g of IRF daily would be very easy and that they felt they could do it, while about 80% responded that it was likely or very likely that if they wanted to, they could easily eat at least 85g of IRF daily, and that it was up to them to do so (Table 3.1). Regarding control beliefs, 38% of girls did not feel able to perform the behavior if they were not able to prepare IRF themselves. About 40% of girls did not feel able to perform the behavior if they were not able to prepare IRF themselves or if they thought it might lead to weight gain (Table 3.1).

**Table 3.1:** distributions (%) of adolescent girls by item on a five-response scale and mean scores (± standard deviation/ SD) on direct and indirect constructs underlying the intention to consume at least 85 g of iron-rich foods per day (N=136)

PBC (direct construct)	Very difficult/unlikely/ strongly disagree	Slightly difficult/unlikely/disagree	Neither easy/likely/agree nor difficult/unlikely/disagree	Slightly easy/likely/agree	Very easy/likely/strongly agree	
For me, consuming at least 85g of iron-rich foods per day would be very easy.	14.0	23.5	3.7	31.6	27.2	0.35 ± 1.45
If I wanted to, I could easily eat at least 85g of iron-rich foods per day.	9.6	5.1	5.9	27.9	51.5	1.07 ± 1.28
I feel capable of consuming at least 85g of iron-rich foods per day.	4.4	5.9	3.7	41.9	44.1	1.15 ± 1.06
It is only up to me to consume at least 85g of iron-rich foods per day.	14.0	8.1	11.0	25.0	41.9	0.73 ± 1.43
All				0.82 ± 0.91		
Control beliefs (indirect construct)						
I would feel able to consume at least 85g of IRF per day even though...						
I think it can make me fat	27.9	16.2	12.5	23.5	19.9	-0.09 ± 1.52
I am not able to prepare IRF myself	30.9	8.1	11.0	19.1	30.9	0.11 ± 1.66
All						0.01 ± 1.13

**Environmental barriers (direct construct):** during FGD, no environmental factor that could promote IRF consumption was identified. Thus, only items relating to environmental factors that could hinder the consumption of IRF were included in the questionnaire (Table 4). The average score related to the environmental barriers was -0.14 ± 0.86, with just over half of the girls (52.2%) answered that it was likely or very likely that the high price of IRF would prevent them from consuming at least 85g daily (Table 4). Between 40% and 50% of the girls responded that it was likely or very likely that the following factors could prevent them from consuming at least 85g of IRF daily: eating with family, IRF are not cooked every day at home, IRF that taste good are not sold near school, parents/guardians do not give them enough money, IRF vendors are far from their school (Table 4).

**Table 4:** distributions (%) of girls on a five-response scale and mean scores (± standard deviation/SD) on environment-related barriers to the consumption of at least 85 g of iron-rich foods per day (N = 136)

Items	% by five-responses scale and scores					Means (± SD)
	Very Improbable (-2)	Slightly improbable (-1)	Neither probable nor improbable (0)	Slightly probable (+1)	Very probable (+2)	
**Do you agree that the following factors could prevent you from consuming at least 85g of rich foods each day?**						
The high price of iron-rich foods	31.6	12.5	3.7	13.2	39.0	0.15 ± 1.75
Eating with the family	33.1	11.0	11.0	16.9	27.9	-0.04 ± 1.65
At home, iron-rich foods are not cooked every day	38.2	12.5	5.1	15.4	28.7	-0.16 ± 1.72
At home, iron-rich foods are not cooked in sufficient quantity	39.7	11.0	11.0	15.4	22.8	-0.29 ± 1.64
Iron-rich foods that taste good are not sold near the school	28.7	17.8	7.4	16.9	29.4	0.00 ± 1.64
My parents/guardians don't give me enough money	33.1	5.1	13.2	16.2	32.4	0.09 ± 1.69
Iron-rich foods that taste good are not sold close to my house	36.8	12.5	9.6	14.0	27.2	-0.18 ± 1.68
Iron-rich foods are sold in dirty places	48.5	6.6	16.9	7.4	20.6	-0.55 ± 1.62
Iron-rich food vendors are far from school	33.3	14.0	7.4	14.7	28.7	-0.12 ± 1.69
Iron-rich food vendors are far from my home	41.2	10.3	11.0	14.0	23.5	-0.31 ± 1.66
All						-0.14 ± 0.86

## Discussion

The consumption of IRF appears to be limited among urban Senegalese adolescent girls [15,39]. In this paper, psychosocial and environmental factors that may influence this eating behavior were explored among girls in two colleges in Dakar, Senegal. Results will be used to define interventions that could improve their consumption of IRF. Overall, adolescent girls reported a strong intention to consume at least 85g of IRF daily. Similarly, girls` attitudes towards this eating behavior were favorable. Moreover, they perceived the SN as being supportive of the consumption of at least 85g of IRF daily. Even though the ATT of a large majority of girls appeared positive in the present study, some negative behavioral beliefs seemed to persist amongst them. Our results concur with Alami *et al*. [40], according to whom most Iranian adolescent girls also expressed positive ATT and beliefs about the benefits of supplement use, while some of them (32%) believed that supplement use was harmful to their bodies and that nutrients should be provided through food. Although their study focused on a different behavior, similar to our context, girls participating in the aforementioned research had a positive attitude towards the increase of their nutrient intakes (either from supplements or foods) likely because of the perceived benefits for their health. In our study, while less than 10% of girls did not believe that consuming at least 85g of IRF daily would make them less tired, prevent complications of future pregnancy, or compensate for blood loss during menstruation, more than a third (34.5%) of girls perceived that consuming at least 85g of IRF daily would make them gaining weight. Likewise, in the study conducted among rural Bangladeshi adolescent girls, Lee *et al*. [41] mentioned that only a minority (30%) were knowledgeable about the link between anaemia and diet.

Many studies have reported that social influences, such as parents, can potentially lead to long-term changes in adolescents' eating intentions and behavior [41-43]. In our research, the SN was positive, and the adolescents´ father and mother were perceived as the people who would approve of the eating behavior to the largest extent. Other studies have also shown that adolescents place a high value on their parents' opinions, especially their mothers, about healthy eating [21-23] which is also the case for our group. The average score on the PBC was low, suggesting that adolescent girls seemed to consider themselves as having a limited ability to perform the behavior. These results contrast with those of Menozzi *et al*. [44], whose findings indicated a strong PBC among Italian students (mean age 22.1 ± 2.6 years) towards the consumption of vegetables. The age difference may explain the disparity in our results, as young adults may have more control than adolescents over their eating behaviors. Also, it should be noted that their study was not conducted in the same geographic and cultural context as ours. In the setting of the current study, girls appeared to have little control over their diet, eating only what their parents or guardians prepared at home or what was offered by street vendors near their school. Although some of them may be involved in preparing meals at home, the choice of what and how much to prepare does not seem to be theirs as also pointed out by Janha *et al*. [22] among Gambian adolescents.

In line with the aforementioned explanation, during FGD, at least 20% of girls reported that they were not able to cook IRF. This potential limitation seemed to influence their perception of control, as almost half of the girls (44.1%) did not feel able to consume at least 85g of IRF daily if they did not know how to prepare them. It is important to note that more than half of the girls (59%) were between 13 and 15 years of age and that in the Senegalese socio-cultural context, adults rather than adolescents are generally responsible for cooking for the entire family. Moreover, in our sample, the average family size was 8.2 ± 3.7, so it may not be obvious and easy for a 13-15-year-old girl to be responsible for cooking for everyone or to occupy any space in the kitchen during meal preparation. In addition, because our participants were going to school, they might be expected to spend more time on their studies than on housework.

In terms of barriers related to the environment, according to participants´ answers, the strongest limitations to consuming IRF were their high price, the fact that IRF that taste good were not sold near the school, and that girls´ parents or guardians did not give them enough money to purchase them. These results are consistent with Verstraeten *et al*. [45] which indicated financial accessibility and taste as major obstacles to the adoption of healthy eating behavior among Ecuadorian adolescents (10-16 years). Similarly, Banna *et al*. [21], reported the lack of financial resources as a barrier to the adoption of healthy eating behavior among adolescents (15-17 years old) in a peri-urban area of Lima, Peru. Lee *et al*. [41] also reported that adolescents were concerned that some nutrition messages, especially those related to the consumption of IRF may not be feasible to implement due to the economic constraints of their households. The affordability of IRF is likely a constraint to their access in Dakar, Senegal. Findings from a study on urban food systems, food security and nutrition in that specific setting have shown that IRF is among the most expensive foods [46]. It is therefore reasonable that adolescent girls' households may have difficulty in accessing them. Moreover, in our sample, about two out of three households were food insecure.

Multiple and interrelated factors, which reflect the environment as well as personal, social, and cultural experiences, influence eating behaviors of individuals including adolescent girls [47]. Interventions to promote healthy eating practices should thus develop strategies that simultaneously address individual, social, and environmental factors [45,48]. The assessment of factors that most likely influence a behavior in a given context is therefore warranted, so that interventions could be specifically targeted to change them. In our context, an intervention aiming at increasing the daily consumption of IRF should target and change certain beliefs about these foods that shape girls' attitudes and the perceived control over the behavior. Environmental barriers such as physical and financial accessibility should also be taken into account.

**Strengths and limitations:** our study has many strengths that should be highlighted. To our knowledge, this is the first research in Senegal assessing individual factors and environmental barriers that may influence adolescent girls' consumption of IRF, using a psychosocial theory to guide the study. Another strength is the development of a questionnaire that specifically aims to assess psychosocial and environmental factors that may influence IRF consumption. Although this questionnaire certainly deserves validation, it is a significant attempt to develop a tool that could be used to collect data on the psychosocial and environmental determinants of IRF consumption in this group. Finally, the limitation regarding our sampling method should be acknowledged: colleges were purposively selected and all girls attending specific classes were invited to participate. Thus, our results may not be generalized to all adolescent girls attending other colleges in the city of Dakar or rural areas.

## Conclusion

This study shows that the overall, urban Senegalese adolescent girls have the intention to consume at least 85g of IRF daily. Yet, the PBC over the behavior as well as environmental barriers appears to limit the adoption of the behavior. In particular, control beliefs persist and likely influence the PCB which, in turn, may impact the intention to adopt the behavior. Keeping that in mind, the next step could be to identify and implement socially acceptable and culturally appropriate behavior change interventions to modify underlying individual beliefs that appear to limit the consumption of IRF. Attention should also be paid to environmental barriers (e.g., financial and physical accessibility of IRF), as these also impact IRF consumption among adolescent girls. Moreover, Senegal's national data collection system could be strengthened to include indicators not only on the nutritional status of adolescent girls but also on their eating behavior and the factors underlying it. This would enable decision-makers to provide targeted responses to the specific nutritional needs of adolescent girls.

### What is known about this topic

- In Senegal, about 2 out of 3 adolescent girls are anaemic, a situation that has been stagnant in the past decade;
- This high prevalence of anemia among adolescent girls in Senegal may be due in part to a diet low in bioavailable iron and/or a diet high in iron absorption inhibitors;
- In Senegal, data on the factors underlying the population's eating behaviors, particularly the low consumption of IRF among adolescents, are scarce.

### What this study adds

- Most urban Senegalese adolescent girls intend to eat IRF;
- A low perceived behavioral control, some negative behavioral beliefs, and environmental barriers such as the high price of iron-rich foods appear to limit the adoption of the behavior.
